# Relationship of size of corpus callosum with white matter changes in elderly population; A retrospective analytical cross-sectional study

**DOI:** 10.1016/j.amsu.2022.104953

**Published:** 2022-11-19

**Authors:** Sadina Shrestha, Bishal Dhakal, Sachin Sapkota, Bishnu Deep Pathak, Binaya Dhakal, Saugat Mainali, Saurav Lamicchane, Nabin Simkhada, Priajan Ale Magar, Sagun Dawadi, Subash Phuyal

**Affiliations:** a Upendra Devkota Memorial National Institute of Neurological and Allied Sciences, Department of Neuroradiology, Kathmandu, Nepal; b Nepalese Army Institute of Health and Sciences, Department of Internal Medicine, Kathmandu, Nepal; c Maulakalika Hospital Private Limited, Chitwan, Nepal; d Jibjibe Primary Health Care Center, Rasuwa, Nepal; e Military Hospital Nepalgunj, Banke, Nepal; f Dhulikhel Hospital, Kavre, Nepal

**Keywords:** Corpus callosum, White matter changes, Magnetic resonance imaging, White matter hyperintensity, Correlation, Elderly, CC, Corpus Callosum, WMC, White Matter Changes, WMH, White Matter Hyperintensity, MRI, Magnetic Resonance Imaging, WML, White Matter Lesions

## Abstract

**Purpose:**

To study the relationship of size of corpus callosum with white matter changes in the elderly population.

**Materials and methods:**

This was a retrospective analytical cross-sectional study. The relationship between the corpus callosum and white matter changes was studied using the magnetic resonance imaging technique, where white matter changes were graded based on Fazekas grading. The Spearman rank order correlation was used to assess the relationship between the size of corpus callosum and white matter changes.

**Results:**

The whole corpus callosum (ρ = 0.165, p = 0.044) and rostrum (ρ = −0.232, p = 0.004) was significantly correlated with white matter changes based on Fazekas severity grading. Similarly, in bivariate regression analysis, white matter changes were strongly correlated with rostrum (standardized β-coefficient = -0.186, p = 0.023). While taking gender in sub-group analysis, white matter changes were significantly correlated with rostrum (ρ = -0.252, p = 0.021) and splenium (ρ = −0.229, p = 0.036) in male and with rostrum (ρ = −0.245, p = 0.048) only in female groups.

**Conclusions:**

Corpus callosum size is associated with white matter changes in the elderly population. This association can give insight into the neuropathology of diseases involving the central nervous system.

## Highlights

• Corpus callosum is the prime fiber tract connecting left and right cerebral hemispheres.
• Thinning of corpus callosum and white matter loss have been shown to occur with aging.
• The MRI has been a vital tool in assessing the changes in brain with age.
• Corpus callosum size is associated with white matter changes in elderly population.

## 1 Introduction

The assessment of the elderly population for various physiological changes is a tedious task. It requires an evaluation from various points and views [1]. With the aging process, some of the associated changes in the brain include white matter changes, cerebral atrophy, and ventricular dilatation [2]. These are the changes described in magnetic resonance imaging (MRI) in absence of a specific disease.

The corpus callosum (CC) is the prime fiber tract connecting the left and right cerebral hemispheres that is integral to the dissemination of information between the two hemispheres. However, age-related changes in CC have been controversial. The majority of the studies have found the age-related thinning of CC. But the thinning has been found only modest in all of them [[3], [4], [5], [6]]. The MRI has been a cornerstone in assessing the changes in the brain with age. And there are different scales (like Fazekas, Scheltens scale, etc.) for assessing those changes in MRI findings [[7], [8], [9], [10]].

As with CC changes, white matter changes (WMC) in the elderly had not been clearly explained in previous literature [11,12]. However, in present settings, white matter volume loss has been shown with the increasing age [[13], [14], [15]]. Similarly, with aging increased signal in the cerebral white matter has been observed in MRI [13,16].

The relationship of white matter changes with the size of corpus callosum the in elderly has not been studied widely. White matter hyperintensity (WMH) in MRI has been observed in various diseases like diabetes mellitus, hypertension, cardiovascular diseases, and even on healthy personnel [17]. In the presence of specific disease like cerebral ischemia and Alzheimer's disease, studies commenting on associations between the size of CC and white matter intensity have been found in the literature [[17], [18], [19]]. But in healthy elderly, this kind of study has been found seldomly.

In this study, we have investigated the relationship between sizes of CC and white matter changes in elderly population using MRI scan. Considering the fact that several studies have illustrated the age-related changes in CC and white matter individually [4,15,[20], [21], [22], [23], [24], [25], [26], [27], [28], [29]], we aimed to assess the relationship between CC and white matter changes in elderly. In contrast to smaller study subjects in majority of previous studies [[17], [18], [19],^25^], we have included considerable patient population to study the relation between CC and white matter changes with the aid of radiological imaging.

## 2 Methods

### 2.1 Study setting, design and participants

This study was conducted in a tertiary care neuro-center of Nepal. The required data for the study was collected from radiology department of the center. It was an analytical retrospective cross-sectional study conducted by reviewing the secondary data of elderly patients. A total of 150 elderly patients were included in the study. The inclusion criteria involved patients with age greater or equal to 70 years and whose imaging information was available. Likewise, the exclusion criteria involved patients with age below 70 years and specific brain related disease like cerebral ischemia, infarction, Alzheimer's disease, tumor, infection, demyelination and large strokes. The study was conducted in compliance with STROCSS guidelines [30].

### 2.2 Data collection and study variables

The required information for the study was retrieved from secondary data that was available in radiology records section. The magnetic resonance imaging (MRI) images were retrieved through picture archiving and communication system (PACS). For the study, variables included were age, length of corpus callosum (CC), genu, body, splenium and rostrum. The white matter changes were graded on the basis of Fazekas grading system [7].

### 2.3 Magnetic resonance imaging (MRI)

A 1.5 T S MAGNETOM Essenza MRI was used for imaging. The Flair images were obtained with repetition time (TR)/echo time (TR), 9000/87 ms; voxel size of 0.9 × 0.9 × 5.0 mm and flip angle 150°. Likewise, T1-weighted sagittal images had TR/TE 500/9.7 ms, voxel size of 1.5 × 0.9 × 5.0 mm and flip angle 90°. The T2-weighted axial images were obtained with TR/TE 400/84 ms, voxel size of 0.7 × 0.6 × 5.0 mm and flip angle 150°.

### 2.4 Ethical consideration

The ethical approval was taken from Institutional Review Committee (IRC-UDM NINAS Reg. No.: 123/2022). The permission was taken from the hospital authority and the chief of record section before starting data collection. The privacy and anonymity of patient information were well-maintained.

### 2.5 Statistical analysis

The statistical analysis was run by using Statistical Package for the Social Sciences (IBM-SPSS), version 25. The normality of the continuous data was checked by using the Shapiro-Wilk test and histogram. Mean/standard deviation and median/interquartile range (IQR) were calculated for normally and non-normally distributed variables, respectively. Frequency and percentages were presented appropriately in tables. The age, length of CC, genu, body, splenium, and rostrum were displayed as continuous variables. The white matter changes grading was displayed as an ordinal variable. The Spearman rank correlation was used to assess the relationship between the size of corpus callosum and white matter changes.

The variables showing significant correlations were taken for multiple linear regression after assessing the possibility of multicollinearity. The corpus callosum size was taken as a dependent variable. Similarly, considering gender, sub-group analysis was performed to study if there was a significant difference between male and female gender. The significance level was taken as p < 0.05, with a 95% confidence interval throughout the analysis.

## 3 Results

The demographic data and MRI characteristics are shown in Table 1. Of the total patients (n = 150), the median age was 78.50 years with an inter-quartile range (IQR) of 73–84 years. The majority of them were males who constituted 56% of the total participants. The mean length of the corpus callosum was 7.25 cm. And the mean thickness of genu, body, and splenium were 8.55 mm, 4.80 mm, and 9.66 mm respectively. Whereas, the rostrum had a median size of 2.70 mm 128 (85.33%) patients out of 150 had white matter changes. But according to the Fazekas white matter changes severity grading, patients having grade 1 changes (42.67%) were more in number as compared to others gradings.

**Table 1:** Demographic and MRI characteristics (n = 150).

Characteristics	No. (n = 150)
Age (years) [median (IQR)]	78.50 (73.00–84.00)
Gender [number (%)]	
Male	84 (56%)
Female	66 (44%)
CC sizes	
CC length (cm) [mean ± SD]	7.25 ± 0.47
CC parts thickness	
Genu (mm)	8.55 ± 1.58
Body (mm)	4.80 ± 0.80
Splenium (mm)	9.66 ± 1.47
Rostrum (mm)	2.70 (2.40–3.10)
White Matter Changes	
No	22 (14.67%)
Yes	128 (85.33%)
White Matter Changes Grading	
0	22 (14.67%)
1	64 (42.67%)
2	44 (29.33%)
3	20 (13.33%)

**Fig. 1:**
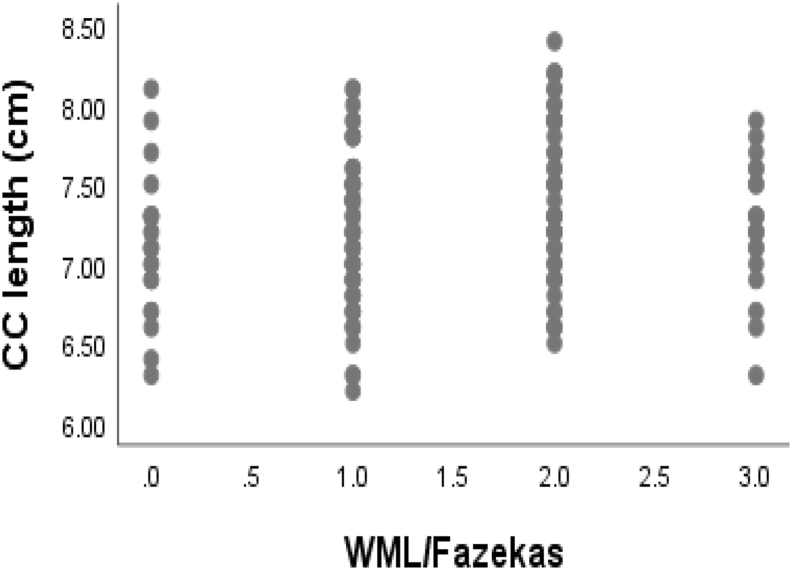
Scatter plot showing correlation between CC length and white matter changes.

**Fig. 2:**
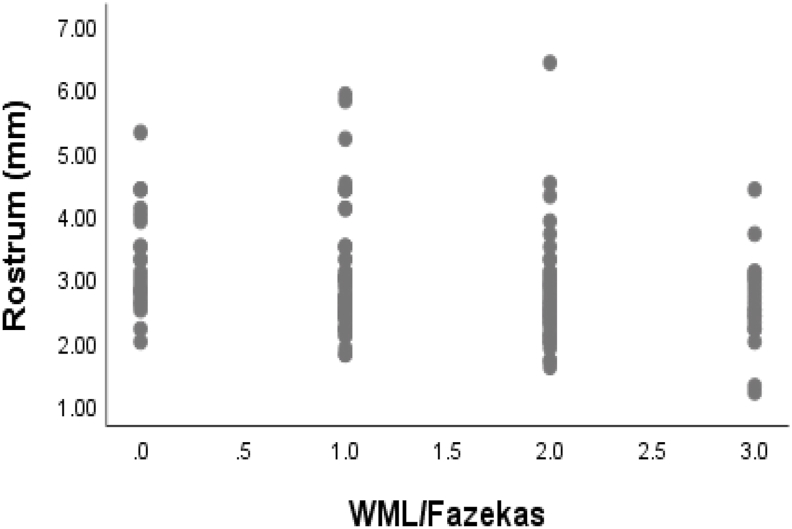
Scatter plot showing correlation between rostrum and white matter changes.

The Spearman rank-order correlation was used to describe the relationship between size of corpus callosum with white matter changes as shown in Table 2. The total corpus callosum was significantly correlated with Fazekas white matter severity grading (ρ = 0.165, p = 0.044). And rostrum was negatively correlated with white matter changes (ρ = −0.232, p = 0.004). However, other parts of CC which were negatively correlated with white matter changes were not significant. The scatter plots showing correlation of total CC length and rostrum with white matter changes are shown in Fig. 1, Fig. 2.

**Table 2:** Spearmank Rank Correlation analysis between corpus callosum sizes and white matter changes.

Variable	Rho (ρ)	P-value
Corpus callosum	0.165∗	0.044
Rostrum	−0.232∗∗	0.004
Genu	−0.106	0.195
Body	−0.138	0.093
Splenium	−0.152	0.064

∗ Significant at the 0.05 level.

∗∗ Significant at the 0.01 level.

In bivariate regression analysis with CC length and rostrum as dependent variables, white matter changes were strongly negatively correlated with rostrum (standardized β-coefficient = −0.186, p = 0.023). Unlike rostrum, the relationship of CC length with white mater changes was not significant (standardized β-coefficient = 0.119, p = 0.148).

Taking gender into consideration, sub-group analysis was done to assess the difference in relation between size of corpus callosum and white matter changes. The rostrum (ρ = −0.252, p = 0.021) and splenium (ρ = −0.229, p = 0.036) size were significantly correlated with WMC in male patients. Whereas, in female only rostrum (ρ = −0.245, p = 0.048) was significantly correlated with WMC. The Spearman correlation coefficients in both of them were negative.

## 4 Discussion

Ageing is a continuous physiological process. And with ageing changes occur in every part of body which may be visible either grossly or by other techniques [1,13]. The age-related changes in the human brain have been described extensively in the existing literature. But in particularly, changes in size of corpus callosum and white matter with ageing are studied for several years [1,6,8,9,13,15,16,[20], [21], [22], [23],^26^,^28^,^29^,[31], [32], [33]]. The atrophy of corpus callosum and white matter changes based on Fazekas grading [7,8] with aging were evident in our study as described in the literature.

The mean length of the corpus callosum in our study patients was 7.25 cm which was comparable with the results by Gnawali S et al. [5] and Takeda S et al. [6]. Similarly, the thickness of the genu, body, splenium, and rostrum were consistent with the findings described by Gnawali S et al. and Takeda S et al. The white matter changes are described as WMH by neuroimaging in our study. Based on the Fazekas WMC severity grading, the grade 1 changes (42.67%) were found more in our study. Similar descriptions have been found in previous studies [16,23,27,34,35] pointing to the age-related degeneration of white matter as evidenced by hyperintensity in MRI scans.

With the considerable study population (N = 150), we found a significant correlation of white matter changes with total CC and rostrum size. However, the significant positive correlation between total CC and white matter changes did not have any significance as there was no any hypothesis to describe such a correlation. The increase in CC size with WMC in the elderly doesn't have any supporting hypothesis till now. But the negative correlation of rostrum with WMC was significant (ρ = −0.232, p = 0.004). It was also evidenced by bivariate regression analysis and scatter plot. This points toward the relationship of CC parts atrophy with WMC in the elderly.

Likewise, the significant negative correlation of rostrum and splenium in elderly males and rostrum only in elderly females with WMC explains the hypothesis suggesting a relationship between atrophy of CC and white matter changes in the elderly.

The findings in our study are supported by previously conducted studies. In a study by Teipel SJ et al. [19] between patients with Alzheimer's disease and normal healthy controls, the correlation was found in healthy controls in contrast to the diseased population. This was supported by another large-scale LADIS study (N = 578) [36] which showed a significant relationship between the area of rostrum and splenium regions and WMC in the elderly. And it explained the association of CC atrophy with increasing loads of age-related white matter changes. However, study by Meguro K et al. [18], Vermersch P et al. [17], and Yamauchi H et al. [25] did not find any significant correlation between CC sizes and WMC in the healthy elderly population. This could be due to the smaller study population.

The relationship of CC sizes with WMC in the healthy elderly population, as established by our study, could be explained due to age-related degeneration in the brain. The significant correlation between them could bring CC changes as an indicator or predictor for WMC in the elderly. With the findings of our study, we are also in accordance with the fact that CC atrophy and WMC are the effect of aging as suggested by Vermersch P et al. [17]. However, some of the small correlation coefficients in the study suggest other possible factors for WMC in elderly individuals. The adequate sample population and significant relation in whole and sub-group analyses stand for the strength of the study.

Like all other studies, we had also limitations in our study. The CC dimensions were measured only in length and thickness. The total CC areas were not measured. Similarly, WMC as indicated by hyperintensity in MRI was not quantified in volumes and according to the different regions. The inclusion of a comparator (diseased) group could have provided the chance for comparison between healthy and diseased elderly.

## 5 Conclusion

CC size is associated with WMC in the elderly as CC atrophy and changes in white matter are seen with aging. This is suggestive of the effect of aging in regions of the brain. And it can predict neuropathological changes that are necessary to explain the neurological disease process.

## Disclosure statement

No potential conflict of interest was reported by the authors.

## Availability of data and materials

The datasets used for the study are available from the corresponding author upon request.

## Registration of research studies

Not applicable.

## Provenance and peer review

Not commissioned, externally peer-reviewed.

## Funding sources

None.

## Ethical approval

The study was approved by the institutional review committee of Upendra Devkota Memorial National Institute of Neurological and Allied Sciences (IRC-UDM NINAS Reg. No.: 123/2022).

## Authors’ contributions

SS contributed in conceptualization, reviewing and editing the manuscript. BD reviewed the essential literature, analyzed the data and drafted the original manuscript. Sapkota S, Dhakal Binaya, SM, SL and PAM collected data and reviewed literature. BDP, NS and SD reviewed and edited manuscript. SP supervised and edited the manuscript. All authors read and approved the final manuscript.

## Guarantor

Bishal Dhakal, Nepalese Army Institute of Health and Sciences, 44600 Kathmandu, Nepal. Email: swarnimdhakal@gmail.com, Phone: +977 9846491651.

## Ethical approval

The study was approved by the institutional review committee of Upendra Devkota Memorial National Institute of Neurological and Allied Sciences (IRC-UDM NINAS Reg. No.: 123/2022).

## Funding

The study did not receive any grant from funding agencies in the public, commercial or not-for-profit sectors.

## Author contribution

We the undersigned declare that this manuscript is original, has not been published before and is not currently being considered for publication elsewhere.

We confirm that the manuscript has been read and approved by all named authors and that there are no other persons who satisfied the criteria for authorship but are not listed. We further confirm that the order of authors listed in the manuscript has been approved by all of us.

We understand that the Corresponding Author is the sole contact for the Editorial process. He/she is responsible for communicating with the other authors about progress, submissions of revisions and final approval of proofs.

**Correspondence:** Bishal Dhakal, Nepalese Army Institute of Health and Sciences, 44600 Kathmandu, Nepal. Email: swarnimdhakal@gmail.com, Phone: +977 9846491651.

Authors as follows:

1. Sadina Shrestha.

2. Bishal Dhakal.

3. Sachin Sapkota.

4. Bishnu Deep Pathak.

5. Binaya Dhakal.

6. Saugat Mainali.

7. Saurav Lamicchane.

8. Nabin Simkhada.

9. Priajan Ale Magar.

10. Sagun Dawadi.

11. Subash Phuyal.

SS contributed in conceptualization, reviewing and editing the manuscript. BD reviewed the essential literature, analyzed the data and drafted the original manuscript. Sapkota S, Dhakal Binaya, SM, SL and PAM collected data and reviewed literature. BDP, NS and SD reviewed and edited manuscript. SP supervised and edited the manuscript. All authors read and approved the final manuscript.

## Consent

Written informed consent was obtained from the patient for publication of this case report and accompanying images. A copy of the written consent is available for review by the editor-in-chief of this journal on request.

## Registration of research studies

Not applicable.

## Guarantor

Bishal Dhakal, Nepalese Army Institute of Health and Sciences, 44600 Kathmandu, Nepal. Email: swarnimdhakal@gmail.com, Phone: +977 9846491651.

## Declaration of competing interest

The authors report no conflicts of interest.
